# Oracular Communications, Addressed to Students of the Medical Profession

**Published:** 1817-09

**Authors:** 


*Oracular Communications, addressed to Students of the Medical Profession.* By Æsculapius. *Octavo, pp.* 131. 1816.

Notwithstanding the *Mythological* mode of ushering in his admonitions, Æsculapius appears to be a well informed man, and not perhaps unqualified for the task which he has here imposed on himself. The Oracular Communications have not the *humour* of Mr. Chamberlayne's Tirocinium Medicum, but they embrace a wide and more dignified scope of observation.

Æsculapius considers, perhaps with justice, that *rectitude of principle, benevolence of disposition, and unwearied diligence,* are more essential requisites for the basis of the medical character, than the possession of shining talents, fortune, or manners. The latter, however, are valuable additions to the former. Without *rectitude of principle,* the practitioner will be too often tempted to look upon the profession as a mere *viaticum to wealth,* and consequently he will be satisfied with obtaining

"*Just so much knowledge of disease and its remedies, as will enable him to pass through life with tolerable credit, and to bury the memory of his blunders in the oblivion of his patient's grave.*"

Whereas, if possessed of *rectitude of principle,* he will daily be striving to add to his store of knowledge, and thus to anticipate the difficulties of practice:—"a chimera little known to the presumption of ignorance."

*Benevolence.* A state of disease is a state of suffering, demanding our sympathy as well as our skill. The mind as well as the body must be medicined. This requires benevolence of disposition; to probe without wounding, and to heal without cauterizing. In obstetrical practice in particular, and in the varied shades of mental disease, from hypochondriasis up to raging mania, benevolence of disposition is essentially necessary. Nor are we to forget the claims which *poverty* has on our humanity and kindness. In the aggravated forms in which diseases present themselves among this class of society, augmented by all the horrors of ignorance and want, it is often in the power of the medical man to smooth the pillow of sickness and the bed of death. The love and esteem of the *poor* are pretty certain indexes of, as well as passports to, success in medical practice; for they prove you capable of that condescension and benevolence which lead you to prefer the relief of human misery to private pecuniary interest.

*Unwearied Diligence.* The acquisition of knowledge is unlimited; and therefore we ought never to rest satisfied with the dull and insipid routine of antiquated practice, but endeavour from each individual case, to elicit information, and add to our general stock.

"*The activity of Intellect* is the very source whence the *power of supporting its exertions* is derived, and every renewed effort is productive of additional strength; hence the vital principle of knowledge is *exertion.*"

Information is always within reach, but it must be sought after.

"The richest talents of the sluggard will be obscured in the rubbish of ignorance, while the moderate abilities of the diligent will be crowned with present success in study, and with future ample accessions of practice." 29.

This diligence must be uninterrupted; not guided by the impulse of a blind attachment to *any part* of the profession in particular; but a determination to obtain all the knowledge that can be acquired, on the ground of its utility in soothing the anguish of disease, and rescuing our fellow creatures from untimely graves.

Our author makes many judicious reflections on the preparatory acquirements and studies of the medical pupil; but for these we shall refer to the work itself. Our extracts and analyses shall be such as are suitable not to the student alone, but the practitioner also.

"Let not Continental medicine be despised or undervalued. There may probably be much in the theory and practice of its professors which will excite a smile, but their patient observation of disease, and discrimination of its symptoms, together with their relation of morbid appearances deserve attention; and there is many a valuable hint to be gleaned from their works. p. 31.

Our author in pressing the value of anatomy upon the student's mind, observes,

"As then, gentlemen, you value your own character and comfort, and the lives and happiness of your patient, cultivate anatomy; *and keep up the knowledge you acquire in London by dissection in the country*, if this be practicable; by comparative anatomy; by reference to books; and more especially by *embracing every opportunity of morbid anatomy.*" p. 53.

But our author sets a proper, and *only* a proper, value on this branch of study, which, though an essential, is in reality but a subordinate item of knowledge. When it is made to preponderate in the scale, the equilibrium of the various classes of scientific and practical information is deranged; and —

"Hence has arisen the universal opprobrium that good surgeons are often deficient in general professional knowledge — that they are unscientific practitioners; their information is greatly confined to anatomy, and to the phænomena and treatment of a few readily cognizable diseases, while they too frequently lose sight of some indirect but collateral considerations of great value." p. 73.

It is not meant by this to undervalue surgical science. If it requires less exertion of thought, yet it affords more decided and immediate relief. The dangers which it averts are readily comprehended; and hence it is always held in great estimation. When we contemplate the tortures and peril of a herniary patient; when we look to the agitation of every bosom in the case of a wounded artery, where another gush may launch our patient into eternity; or when we reflect on the awful state of an aneurismal patient, with a large tumour pulsating under our fingers, and rapidly hastening to that state when the external covering will give way, and the victim be hurried to the grave; when we recollect that in all these, and in numerous other cases, the surgeon steps in and rescues his patient from present suffering and impending destruction, it is impossible to think lightly of SURGERY!

But a good surgeon must be a good physician. Surgical diseases produce constitutional, which require medical treatment; and nobody now questions the constitutional origin of many local complaints.

"Never take into the account, the opinion of the world: let your decisions be the result of deliberate judgment, and act upon them firmly, undeviatingly, satisfied with possessing, under every circumstance, the *mens conscia recti* of having consulted only your patient's good, and of having dismissed the paltry considerations of *self-interest*." 81.

*Physic.* It is a common error with professional students to neglect physic; and yet medical knowledge can alone make a good and successful practitioner in surgery. It is, besides, demonstrable, that for one surgical, we meet with twenty medical cases; and although a successful operation is highly gratifying, and profitable to a young practitioner, yet, how comparatively insignificant is this consideration, when contrasted with the power of relieving those every day sufferers who are groaning under the pressure of disease which no eye can see—no scalpel reach! The value of the two sciences must be estimated, not by the display occasioned by their exercise, but by the positive alleviation afforded, and by the frequency of the recurrence of occasions demanding this relief.

"And here, gentlemen, I must be permitted to caution you against blindly adopting any system of opinion or practice which may be taught in the schools to which you may be respectively, and more immediately attached; this would be to degrade you to the ranks of empiricism. Think for yourselves. It generally happens, that systems are carried to extremes by their authors; and that, however valuable and excellent they may be in the hands of judicious practitioners, they will invariably prove hurtful when acted on by those who embrace the dogmas of the profession without thought, and without discrimination; and who in the loneliness of their minds, find only one uniform spectral impression; even the illusion occasioned by the corruscations of genius, perceived through the vapour of a clouded understanding, and reflected upon an eye already tinged with the morbid hue of prejudice, already distorted by the obliquity of the judgment. Be it yours, gentlemen, to judge, to examine, to determine for yourselves: listen to the system of your Lecturer, adopt it; act upon it; but preserve your independence, and be not enslaved by it. Enlarge your minds by perusing other systems, and contrasting these with that you have been more immediately taught; and amidst every contradictory and favourite hypothesis, maintain your own liberty of thought and action, dismiss the prejudices of education, and bring the decisions of your cool and INFORMED judgment to the test of experience.

"Let your conduct be the same with regard to medical practice; recollect, in fact, that ye are men of intellect; you go to learn, not to imitate; you seek instruction, and not a mechanical capacity of writing a prescription. Allow me to introduce one other caution. Do not expect to meet with disease at the bedside, as you see it at the theatre, or in your study. Here you are presented with abstracted cases of well defined disease; cases in which every symptom not necessarily pertaining to specific morbid action is omitted. But in practice, this is comparatively rare; and hence you will frequently be at a loss how to define and classify disease; and if you be a systematic practitioner, you will scarcely know how to direct the appropriate remedy; since you have been accustomed to connect a regular train of symptoms with certain constant organic lesions, and these have hitherto been associated in your minds with a certain class of materia medica. But it will not do, gentlemen, it is first principles you have to acquire; it is the soul of physic you have to obtain. Practice and reflection will build up the body to maturity, and make the perfect man. Be therefore greedy of medical lore, and steal every half hour you can obtain for this important purpose." p. 94.

If health be a blessing, disease an evil, or benevolence a virtue—if it be an object to alleviate the wretchedness of others, and to restore to them those comforts which health alone can enable them to enjoy, then it is infinitely important for those who practice the profession, to be most intimately acquainted with it; and, dismissing the selfish argument of pecuniary reward, there cannot surely be a more honourable pursuit than that which secures respectability and influence—the great ends of living, and will be attended with pleasures which it is hardly possible to appreciate or define, since they are those which a sensible mind enjoys in the discharge of a duty, and in the exercise of beneficence. If, however, we neglect to cultivate our knowledge by the most unremitting attention to the phænomena of disease, we shall assuredly prepare for ourselves a pillow of remorse, while an unavailing and cruel regret may embitter our future life!

The following "Oracular" admonition, which will conclude our extracts, is not unworthy of the son of Apollo to deliver, nor of the Machaons and Podalirii of these times to receive with due deference and respect from the Æsculapian sage.

"Let me again caution you against supposing, that the acquisition of information is to cease with your leaving the hospital; I will repeat, that a life will be *well* spent in pursuing medical science. Your studies must know no termination; knowledge must be ever advancing. With any other views than these, you will degenerate into the mere practitioners of habit, and arrive at all the honours of empiricism. Let me remind you of the increasing responsibility incurred by successive years; and as each rolls away, it behoves you to have added to your knowledge and power to heal. It is unfortunately a too frequent occurrence, that practitioners are contented that their knowledge should remain stationary: they embrace a certain system, and in the sphere of its attraction, they move as mechanical bodies guided by external impulse: they are carried through life by the machinery of time, and they revolve in the same dull and endless round, without thought, without vitality. They are heedless of the improvements that are going on around them: they do not pretend to benefit by experience; and their practice is the same with all its errors and imperfections, as when they commenced their career, deformed with all the obliquities of imperfect conception, heightened by prejudice and erroneous association. They give up the use of reason; their actions automatic and invariable. But, gentlemen, as you value the profession you have embraced, avoid this undeviating pursuit of one beaten track, and find a path for yourselves. Do not refuse indeed to pluck a rose from the accustomed path, but do not reject or despise the fragrancy of a wild flower which may grow at some distance from the road side. I would studiously avoid giving the smallest encouragement to the sceptical experimenter, who sports with the lives and health of mankind at the mere bidding of caprice; but surely it is possible to avoid the one danger, without being exposed to the influence of the other. Go forward, gentlemen, determined to add to your stock of information, and to render every accession to your store available for your own comfort, and the good of your patients. Study only and simply with *a view to relieve*, and you will be preserved from any wanton or unnecessary experiment. Let experience be valuable to you. Collect and arrange your facts, and by their multiplication at last draw your inferences. Be not slaves to the monstrous insipidity of the *recurrence of disease and remedy*; but think for yourselves, and adopt the improvements suggested by conscience, or enlightened by judgment.

"For this purpose, let me earnestly advise you to take daily notes of all that is interesting in your practice. You will thus accumulate facts, and gradually become possessed of a multitude of data from which to establish your rules of practical prudence. Many advantages will accrue from this practice, not only in obliging you to think over, and to take notice of what is passing, but in inducing a habit of reasoning and reflection, teaching you to review the past, and to draw legitimate inferences for the regulation of the future. You will not find this to be lost time, but on the contrary, most profitably spent. I do not indeed recommend, nor can I approve the formidable array of a "note book," when you visit your patient. This may be very right in hospital practice, but it wears the appearance of ostentation in private, and moreover frequently excites uneasiness on the part of the patient, from the impression of there being somewhat extraordinary in his case. But of an evening, review the past day, and commit that which is worth recording to paper. Adopt this plan, and you will speedily experience its beneficial consequences.

Lastly, gentlemen, one word on the subject of reading: books to the medical man, are as necessary as his daily food; and ought to be a source of heavy expenditure. Reading can alone make the perfect man. The science of medicine is daily improving, knowledge is constantly advancing, and yours should keep pace with the progression of science. Never be contented to remain stationary, but be ever aiming after greater acquirements. Till you get into practice, you will probably not have read much. Now, therefore, is your time, when you have gained that solid information which will enable you to separate the good from the bad, and to retain only that which is valuable. There is much folly, and sophistry, and misrepresentation to be met with in medical writers. But there are few books that are not worthy your perusal, and from which some information may not be extracted; this, however small, will be an ample compensation for the labour of perusing the work, even though it should contain many a dull and stupid page; many a groundless hypothesis; many a learned dogma; many ill-grounded conclusions. But you will perhaps say, that time cannot be found for this pursuit. I reply that it may, if you are anxious to obtain it, and if you avail yourselves of its presence. You must learn the secret of making time. This is an invaluable art. Learn to improve and employ the shreds of time, and diligently to exhaust every atom as it passes. Avoid those habits of wasting your hours, which you may fall into from the want of order and system. Devote some part of the day to this professional duty; and if no other can be found, set apart a certain proportion of the hours usually devoted to sleep. Believe me, Gentlemen, it is not difficult to create time; for whatever is really pleasing to you, and the excuse of *want of time*, is generally, but another and a gentle name for indifference and disinclination. It is a paramount duty to increase and keep up your knowledge; nor can this be effected by simply taking in a medical periodical work. Become a literary character, a *gourmand*, if you will, but read authors for yourselves, and *digest them*. Nor let your reading be confined to a few favourites; but take an enlarged view, peruse the enemies as well as the friends of your darling system, and be open to conviction. Take a comprehensive view of disease: enlarge your views by the study of foreign authors; devote your whole heart, your life, to professional pursuits. So shall you find pleasure in the performance of duty, and an ample reward for every toil. On this subject I cannot say too much, but I forbear, believing that enough has been advanced, to induce you to prefer the pleasures of intellect to the gratification of the passions, and to lead you to substitute *literary pursuits* for the sports of the field, the pleasures of social conviviality, or the mystic delights of whist." P. 131.

Notwithstanding the veil of *antiquity* which Æsculapius has thrown round him, and the anonymous obscurity with which these *oracles* are delivered, we can easily descry in their author the effusions of *youth,* rather than the dogmas of age. Æsculapius has tendered good advice both to the student and to the practitioner. On the mind of the *former* it is more likely to make impression than on that of the latter, and to *him* we warmly recommend it. In Æsculapius we recognize a mind ardent almost to enthusiasm, in the cause of medical science; and, as yet unchilled, undisgusted by the rude blasts, the bitter disappointments, which it is destined to encounter in the subsequent voyage through life. May he prove one of those few gallant souls who rise with undiminished elasticity against every pressure—who, in pursuit of knowledge, brave the "whips and scorns of time," and cheerfully sacrifice luxury and pleasure at the shrine of Hygeia, and of honourable distinction in the noblest of arts, the most useful of sciences!

---

### PART III.

### SELECTIONS.

—— Factorum est copia nobis.

